# Unsung Heroes: How Senior Centers Have Adapted to Provide Essential Services in the Face of COVID-19

**DOI:** 10.1093/geroni/igab046.1229

**Published:** 2021-12-17

**Authors:** Ceara Somerville, Jan Mutchler, Caitlin Coyle

**Affiliations:** University of Massachusetts Boston, Boston, Massachusetts, United States

## Abstract

This presentation will describe the ways in which senior centers in Massachusetts have adapted during the COVID-19 pandemic. Three surveys (distributed in April, August, and November, 2020) were conducted with 342 senior centers in the state to learn about current operations through the pandemic, challenges faced, and steps taken to solve those challenges. Results suggest that almost all senior centers (91%) continued to provide limited programming or essential services during the pandemic. Senior centers are prioritizing socialization and nutritional needs as critical services, but are changing the way they operate to continue to meet those needs. Despite facing uncertainty about the future, senior centers continue to adapt to changing conditions as they seek to meet their core mission. This presentation will discuss effects of COVID-19 on how senior centers will continue to operate through and post-pandemic times as well as local and state policy implications.

